# Examining the historical trend of wind turbine maximum capacity in Egypt

**DOI:** 10.1038/s41598-025-24866-z

**Published:** 2025-11-23

**Authors:** Mohamed Tarek Sobh, Mohammed Magdy Hamed, A. O. Elgharib, A. R. El-Mallawany

**Affiliations:** 1 Construction and Building Engineering Department, College of Engineering and Technology, Arab Academy for Science, Technology and Maritime Transport (AASTMT), B 2401 Smart Village, 12577 Giza, Egypt; 2 Basic and Applied Science Department, College of Engineering and Technology, Arab Academy for Science, Technology, and Maritime Transport (AASTMT), B 2401 Smart Village, 12577 Giza, Egypt; 3 Mechanical Engineering Department, College of Engineering and Technology, Arab Academy for Science, Technology and Maritime Transport (AASTMT), B 2401 Smart Village, 12577 Giza, Egypt

**Keywords:** Sen’s slope, Egypt, Wind energy, ERA5-Land, HAWT, Modified Mann-Kendall, Civil engineering, Electrical and electronic engineering, Mechanical engineering

## Abstract

Wind energy is a clean, consistent, and increasingly affordable renewable energy source that plays a crucial role in addressing the challenges faced by fossil fuel resources. Due to Egypt’s unique geographical position and elevated mean wind speeds, Egypt has become a front-line player in the world wind industry. Egypt’s coastal regions, particularly along the Red Sea and Mediterranean coastlines, possess an unmatched potential for wind power, making these areas highly favorable to large-scale wind farms. Egypt’s wind power potential is examined through a thorough mapping and in-depth analysis of the distribution parameters of wind speed based on ERA5-land reanalysis data. Zafarana, Egypt’s primary wind energy center, has provided the opportunity to install numerous Horizontal Axis Wind Turbines (HAWT). The current study refers to other regions’ potential, especially in southern Egypt, where wind speed has experienced an impressive rise of 0.2 m/s over recent decades. As part of presenting an exhaustive explanation of Egypt’s wind power dynamics, this research samples nine representative wind turbines, divided into two distinct periods: the earlier period (1950–1979) and the more recent period (1990–2019). By comparing these two phases, the study discovers how climatic conditions and technological advancements have affected wind turbine efficiency and performance. The maximum capacity ratio of the 15 MW wind turbine is in the range from 38% to 43%. The findings of this study not only show the enormous potential of wind energy in Egypt but also highlight the value of strategic investment and planning in untapped regions. Such a study provides a foundation for policymakers, researchers, and industry stakeholders to optimize wind power utilization and enhance Egypt’s overall renewable energy targets. Lastly, the effort assists in enriching the country’s ability to limit global warming as its economic progress is improved by establishing its abundant wind resources.

## Introduction

Due to technical improvements, electrical energy output increases between 4 and 8% annually^1^. Fossil fuel reserves, crucial in supplying the increasing energy demand, are running out remarkably. It is clear that these reserves will soon run out and will not be enough to meet customer demand^2,3^. The growing interest in renewable energy sources as a substantial replacement for existing energy sources results from the finite supply of fossil fuel resources and environmental problems caused by air pollution and the greenhouse effect^4^. Over the last five years, wind power has grown at an average annual rate of 34% worldwide^5^. Wind is an electric power source and a renewable energy technology with the fastest growth rate. Promoting sustainable energy resources and systems to increase the share of renewable energy of Egypt in electricity generation to 40.3% based on The National Agenda for Sustainable Development^6^. A spatial assessment of wind power potential at a global scale showed that Egypt has great promise in terms of developing its electric sector and has high wind power density compared to other African countries^7^. The assessment categorized the wind power densities all over the world into seven categories, and considered Egypt in the middle category.

Derived from the sun, which is abundant and freely available in the environment, wind energy is a safe and uninterrupted energy source^8,9^. Variations in temperature and pressure brought on by the unequal distribution of solar energy across the Earth’s surface produce wind. When air moves from a high-pressure area to a low-pressure area concerning the Earth’s surface, it is called wind^10^. Industrialized and developing countries actively embrace wind energy as a new energy source. Using this energy to build wind power plants is becoming increasingly popular worldwide. In recent years, there has been a surge in wind energy projects, with many countries possessing sufficient wind resources to meet electricity demand. However, achieving electricity sustainably requires rapid infrastructure development, such as grid modernization, energy storage systems, and policy frameworks.

Due to the growing need for renewable energy, wind energy is essential in the global shift to sustainable energy solutions^12^. Onshore and offshore wind turbines are now central to harnessing wind power for electricity generation, with studies analyzing their output power densities across diverse geographic and climatic conditions. Wind turbines are constrained by two primary challenges: their inherent intermittency (due to variable wind patterns) and the difficulty of identifying optimal wind sites, particularly in developing regions^13^. Research demonstrates that interconnecting multiple geographically dispersed wind farms can mitigate intermittency by smoothing out localized fluctuations in wind speed^14^. This strategy leverages the reduced spatial and temporal correlation of wind speeds across larger areas, enhancing the reliability of wind power as a renewable energy source.

Wind turbines transfer wind energy into mechanical power, which is converted into electrical power. Power density, which is the quantity of electrical power produced per unit area of the turbine’s swept area, is commonly used to quantify the performance of these turbines^15^. Understanding turbine performance is essential for determining the feasibility of wind energy projects. According to^16^, a network of wind farms spanning portions of Europe and Northern Africa could provide almost 70% of Europe’s electricity needs. Wind electricity is predicted to cost less than 5 cents per kWh, even after accounting for transmission and storage expenses. Avoiding long-distance transmission line travel is a top concern^17^. It is well known that combining transmission lines from several geographically separated wind farms to generate electricity lowers output variability^18^.

Egypt’s power generation comes only from natural gas (divided into 77.37% natural gas and 12.64% other fossil fuels), with 6.49% hydroelectric power and 3.6% renewable energy resources^19^. A step towards sustainability, Egypt focuses on meeting the growing energy demand using clean energy technologies^20^. Egypt has set a target to reach 42% of total capacity from renewable energy resources by 2035^19^. Wind energy has a great potential as high wind speeds were concentrated in Egypt’s center, which gradually increased to the maximum in the central South^21^. Though Egypt has excellent potential for wind energy, especially in the Gulf of Suez and along the Red Sea coast, various obstacles have delayed its full-scale development and operating efficiency^22^. The lack of long-term meteorological data has impeded Egypt’s wind energy projects since accurate resource evaluation and project planning depend on it. Environmental variability affects output since wind speed and consistency vary around the region^22^. Economically, securing regular financing remains challenging, especially for large projects that require substantial upfront costs. Economically, the significant upfront costs of wind energy projects provide major obstacles. Project delays and higher fees can result from obstacles, including environmental impact evaluations and complex licensing processes^23^. Despite the technical promise, these interacting elements have reduced Egypt’s capacity to maximize wind energy efficiency.

The present study focuses on the potential of wind energy in Egypt while considering historical climate conditions. This study focuses on the meteorological conditions of Egypt from 1950 to 2019. Both wind speed and mean temperature were divided into two periods. Period-1 (P1) and Period-2 (P2) from 1950 to 1979 and 1990 to 2019, respectively. While considering the previous meteorological conditions, nine wind turbines were studied across Egypt using the ECMWF Reanalysis v5-Land (ERA5-Land) dataset. The nine wind turbines have different output power capacities ranging from 0.1 MW to 15 MW. This study aims to suggest new and different locations for wind farms across Egypt.

## Study area

**Fig. 1:**
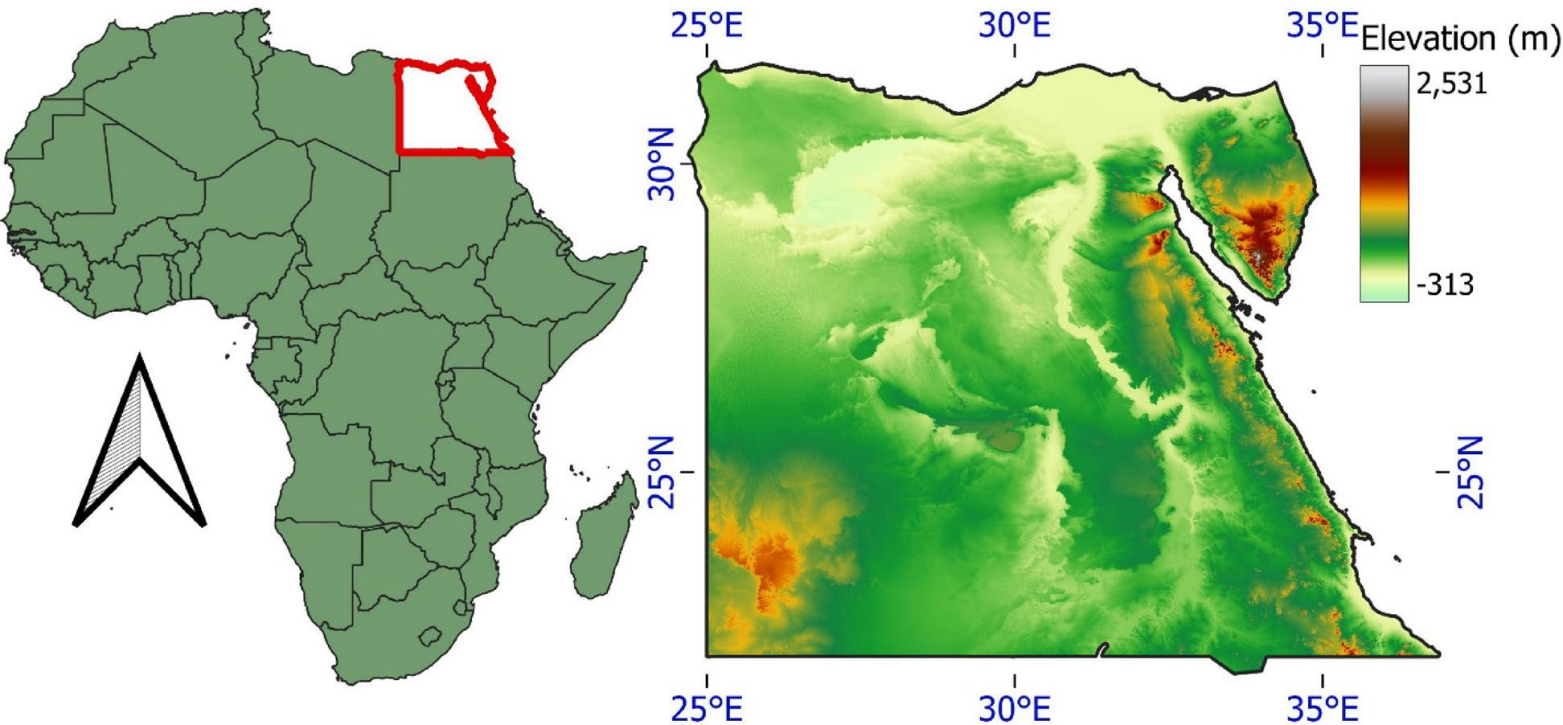
Study area location on Africa’s map and the elevation map of Egypt (The figure generated using Qgis V3.40 https://qgis.org/download/).

Egypt’s diverse topography, shaped by deserts, plateaus, and the Nile River, is critical in determining its wind energy potential^24^. The topography is primarily flat except for a few plateaus and mountainous locations. Figure 1 shows the location of Egypt on Africa’s map and the elevation map of Egypt. The Nile River is one of Egypt’s most important topographical features. The river flows through the nation for more than 4,000 km before draining into the Mediterranean Sea. Unlike the stony and barren desert areas on either side of the river, the Nile Valley is a rich strip that runs parallel to the river. Mount Catherine, on the Sinai Peninsula, is Egypt’s highest point, at around 2636 m. The mountain has a variety of rock formations as well as unusual plants and animals. The high elevations affect air properties, which will affect the captured energy by the wind turbines^25^. In addition to its geographical features, Egypt has a well-defined environmental policy to support wind energy development. The government’s Integrated Sustainable Energy Strategy (ISES) 2035 targets 42% of electricity from renewables, with wind power being a key component^26^. Despite existing wind farms in regions like Zafarana, different areas of Egypt remain underexploited for wind energy. These established projects, such as those in the Gulf of Suez, demonstrate a proven capacity for large-scale wind infrastructure and attract significant foreign investment^27^. For instance, Dakhla South in the Western Desert exhibits promising wind speeds, ranging from 4.4 to 6.3 m/s at 10 m height and 5.4 to 7.7 m/s at 24.5 m height, indicating strong potential for utility-scale wind projects^28^. Such data highlights the need for comprehensive site assessments to leverage Egypt’s topographical diversity for sustainable energy production.

## Data used

Weather station data in Egypt is limited by sparse spatial coverage, data gaps, and quality problems, making it insufficient for comprehensive wind and temperature analysis. Reanalysis datasets like ERA5 or ERA5-Land offer a reliable alternative with high-resolution, long-term, and spatially consistent data across the country. The ECMWF’s fifth-generation reanalysis (ERA5-land) provides a variety of global atmospheric, land surface, and sea state data with a resolution of 9 km^29–31^. ERA5-Land integrates the model data with observations from around the world using 4D-Var data assimilation and model forecasts in Cycle 41r2 of the ECMWF Integrated Forecast System to provide a consistent and comprehensive global dataset^32^. Global hourly estimates for a wide range of atmospheric, ocean wave, and land surface variables are supplied by the ERA5-Land reanalysis data, which spans the years 1950 to the present. In this study, the hourly u and v components of the wind speed were used along with the temperature across Egypt. The hourly data from 1950 to 2022 were obtained from https://cds.climate.copernicus.eu/datasets/reanalysis-era5-land and converted to the monthly scale. ERA5-Land reanalysis data was chosen over other reanalysis datasets because it has a higher spatial resolution (0.1°), temporal resolution (hourly), and temporal coverage (1950-present).

Researchers across diverse disciplines have recently leveraged the ERA5/ERA5-Land dataset to advance their studies. A comparative analysis confirmed that ERA5-Land is reliable for evaluating wind resources^33^. compared the ERA5 wind speed to the global sub-daily dataset (HadISD). From climatological and renewable energy perspectives, hourly wind data from ERA5 reanalysis is valuable information for further detailed studies requiring consistent spatial and temporal wind distributions^33^. In a subsequent study on weather conditions in the United Kingdom^34^, evaluated hourly 10-meter ERA5 wind speed data against in situ measurements from 205 onshore and offshore meteorological stations. The results revealed that, although some biases and errors were present, the overall performance of ERA5 (assessed through mean wind speed bias and root-mean-square error (RMSE)) was superior to other global reanalysis products within the UK domain, outperforming findings reported in earlier studies. Although directly using ERA5 reanalysis is strongly discouraged when addressing very heterogeneous sites regarding land use, resulting in highly changing roughness conditions^35^. ERA5 scores are generally acceptable in Egypt, at least for wind speed, due to its homogenous land structure.

Beyond wind data, ERA5 has also shown strong performance in temperature modelling^36^. validated ERA5-Land temperature data using 12 automatic weather stations across Brazil, reporting RMSE values below 0.60 °C, indicating high agreement between reanalysis and observed temperatures. Similarly^37^, assessed ERA5 temperature estimates over Turkey for 1951–2020, comparing them with trends derived from ground-based station data. The study found high consistency between ERA5 and observed temperature trends, suggesting that ERA5 can be a reliable substitute for observational data in specific contexts^38^. evaluated the ERA5 data by comparing it with land-based data obtained from weather stations on the global historical climatology network (GHCN) across two locations in Dublin and Singapore. The results showed that ERA5 data is most reliable when measuring mild temperatures^38^. These studies suggest that ERA5-Land reanalysis provides robust estimates of near-surface air temperature, making it a viable alternative to in situ measurements when observational data are missing or sparse.

## Methodology

**Fig. 2:**
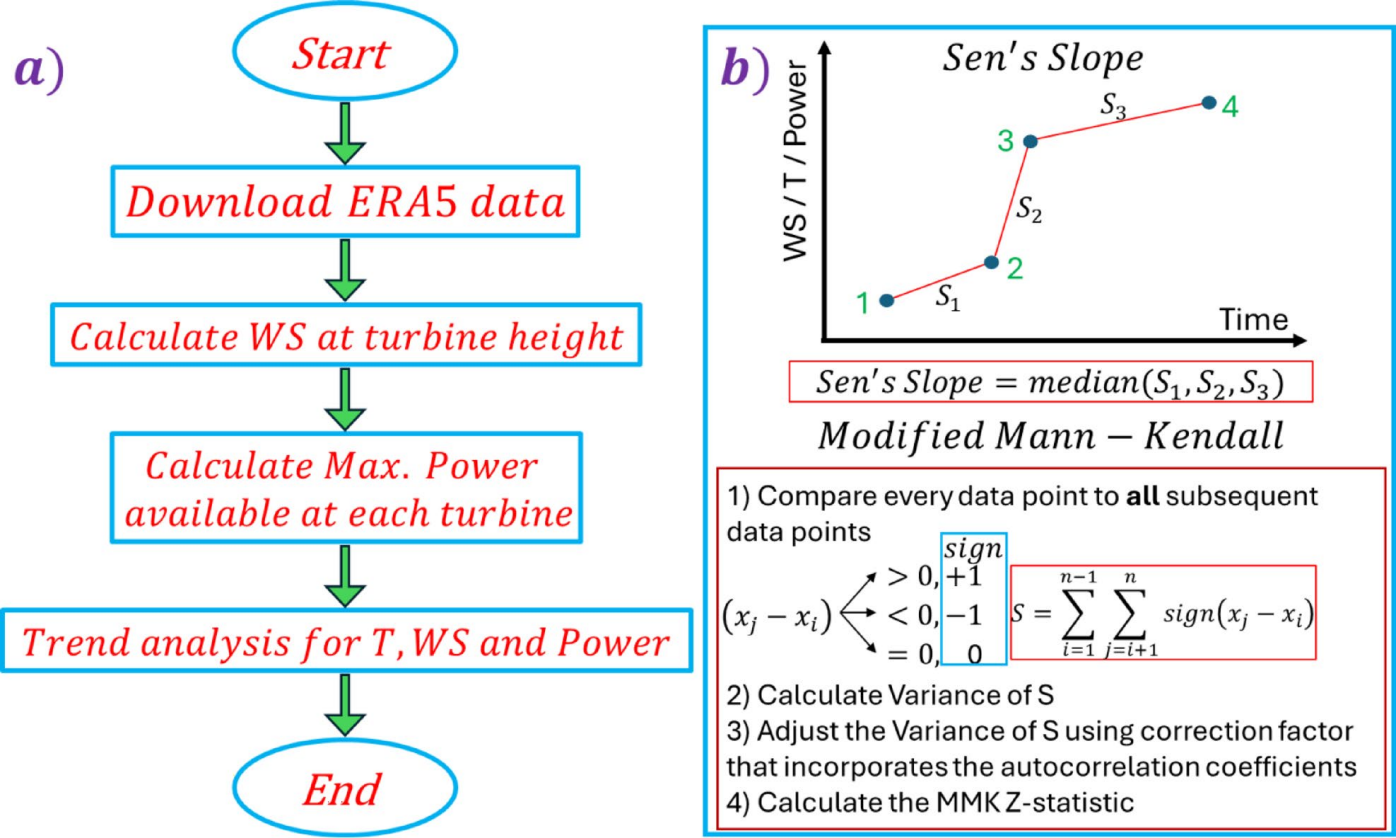
**(a)** flow chart of the methodology implemented and **(b)** detailed methodology on Sen’s slope and MMK.

The present study suggests a suitable location for wind turbines and a suitable Horizontal Axis Wind Turbine (HAWT) for each location. ERA5-land provided the weather conditions of Egypt. The study focuses on the period from 1950 to 2022, which is divided into two periods. The first period is from 1950 to 1979, while the second is from 1990 to 2019. The ERA5-land provides the wind speed and mean temperature across Egypt. The wind speed data (u and v) were at an elevation of Z (10 m). The magnitude of the u and v components was calculated by Eq. 1 for each point. Sen’s slope estimator quantified the magnitude of the change (MW/decade) to assess long-term trends in wind power potential, while the modified Mann-Kendall^39^ test determined the statistical significance of these trends as shown in Fig. 2. The atmosphere’s boundary layer is affected by turbulence at all altitudes, and topographical variation of the change with height and surface roughness. As a result, the average speeds increase with increasing altitude. All values of the wind speeds were calculated at different heights while considering the tower height ($\:{Z}_{R}$) by Eq. 2.

1 $$\:V\left(Z\right)=\:\sqrt{(u^2+\:v^2\:)}\:$$

Where $\:V\left(Z\right)$ is the wind speed available at a height Z (m/s), and $\:u$ and $\:v$ are wind speeds in x and y directions (m/s), respectively.

2 $$\:V\left({Z}_{R}\right)=V\left(Z\right)\left(\frac{\text{ln}\left(\frac{{Z}_{R}}{Zo}\right)}{\text{ln}\left(\frac{Z}{Zo}\right)}\right)$$

Where, $\:V\left({Z}_{R}\right)$ is the wind speed at a height $\:{Z}_{R}$ (m/s), and $\:Zo$ is the roughness height (m).

### Wind turbine model

The key elements of wind energy conversion systems are the generator, aerodynamics, actuator pitch, power converter, and control part. The maximum estimated extracted power by the turbine was calculated by Eq. 3. The power available in an airflow is not totally converted by the wind turbine; therefore, the efficiency of wind turbines is quantified by the power coefficient. The estimated output power was calculated daily and monthly while considering the Betz limit^40^. This limit is the ratio of the extracted power by the turbine to the total available power of the wind airflow. The density of air was calculated while considering the elevation of the turbine above the mean sea level (Eq. 4). The capacity ratio, which is the ratio of the maximum power available to the rated power of the wind turbine (as shown in Eq. 5), was applied to the nine turbines presented in this study.

3 $$\:{P}_{tmax}=\frac{1}{2}\:{\uprho\:}\:{A}_{T}\:{V}^{3}\frac{16}{27}$$

Where, $\:{P}_{tmax}$ is maximum power available (W), $\:{\uprho\:}$ is density of air (kg/m^3^), $\:{A}_{T}$ is the cross-section area (m^2^), and V is wind speed (m/s).

4 $$\:{\uprho\:}=\:\frac{353.049}{T}{e}^{(-0.034\:\frac{elev}{T})}$$

Where T is the temperature (ºK), and elev is the elevation of the turbine above mean sea level (m).

5 $$\:C=\frac{{P}_{tmax}}{{P}_{R}}$$

Where $\:C$: Capacity ratio (%), $\:{P}_{tmax}$ is the maximum power available (W), and $\:{\varvec{P}}_{R}$: rated power of wind turbine (W).

### Wind turbines specifications

Nine different wind turbines from T-1 to T-9 have been utilized, as shown in Table 1. The largest wind turbine (T-1) used in this study, which can produce the rated output power of 15 MW, has a tower height and radius of 166 and 135 m, respectively. The smallest wind turbine (T-9) can produce a rated output power of 0.1 MW and has different dimensions from T1, with a tower height of 24 m and a radius of 10 m, respectively.

**Table 1:** Wind turbine specifications.

Name	Power (MW)	H (m)	*R* (m)
T-1	15	166	135
T-2	10	140	96.5
T-3	5	100	65
T-4	2	80	40
T-5	1.5	80	38.5
T-6	1	60	27.1
T-7	0.75	50	23.5
T-8	0.225	40	14.5
T-9	0.1	24	10

### Trend analysis

Sen’s slope estimator (Sen, 1968) is used to quantify the magnitude of change, while the Modified Mann-Kendall (MMK) test (nonparametric method) was employed to assess the statistical significance of trends. The MMK test has been widely recognized as a more robust and reliable alternative to the original Mann-Kendall (MK) test for detecting trends in meteorological data, particularly in serial correlation^41–43^. This method is minimally affected by outliers, does not assume a specific data distribution, and can adjust missing values. The MMK test was selected to mitigate the potential influence of autocorrelation on trend significance. When applied to a time series, Sen’s slope calculates the slope between each pair of consecutive data points and then determines the median of all these slopes to estimate the overall trend magnitude^44,45^.

## Results

**Fig. 3:**
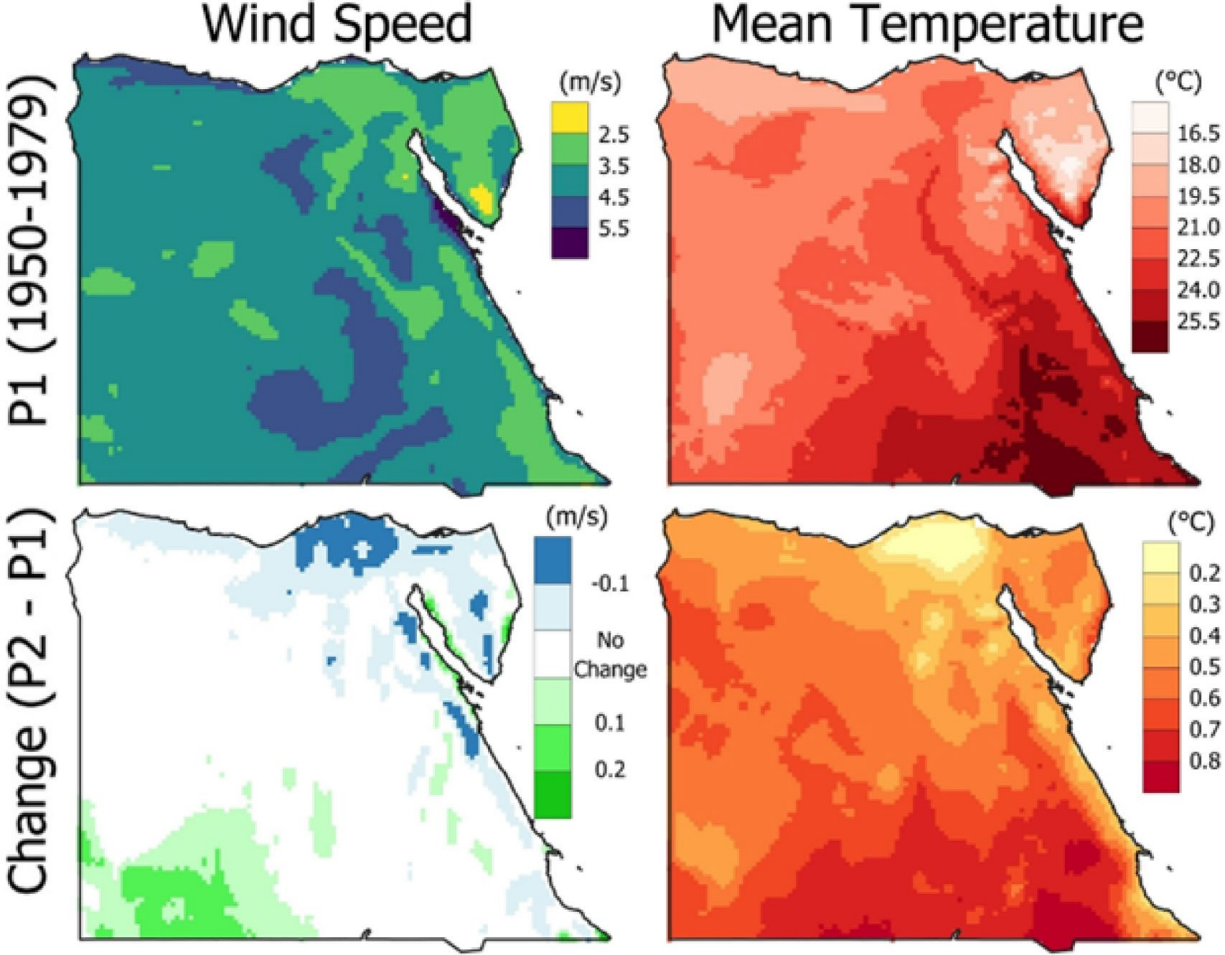
Spatial distribution of wind speed (left column) and mean temperature (right column) from 1950 to 1979 (top row) as a reference period, and the absolute change (bottom row) from 1990 to 2019 with the reference period (the figure generated using Qgis V3.40 https://qgis.org/download/).

The historical spatial distribution of both wind speed and mean temperature in Egypt is presented in Fig. 3. The first row represents the reference period (P1) from 1950 to 1979, while the second row shows the absolute change from 1990 to 2019 (P2) from the reference period (P2 - P1). The wind speed ranges from 2 to 6 m/s, and the mean temperature ranges from 16 to 26 °C. The highest wind speed was observed at Ras Gareb (> 5.5 m/s), while the lowest wind speed was in South Sinai. Most of the country had wind speeds ranging from 2.5 to 4.5 m/s. Regarding the change in wind speed, the highest increase was observed in southwest Egypt with more than 0.3 m/s, while the northern region faced a decrease of −0.2 m/s. Ras Gareb faced an increase of 0.25 m/s compared to the reference period, while South Sinai decreased by −0.1 m/s. The speed in most regions remained unchanged compared to the reference period. The mean temperature across most of Egypt ranges from 19 to 22 °C. The highest and lowest mean temperatures were observed in the southeast of Egypt and South Sinai, respectively. The highest and lowest change in the mean temperature was observed in the southeast and north of Egypt, respectively. A change in the mean temperature range of 0.4 to 0.6 was observed for most of the region.

**Fig. 4:**
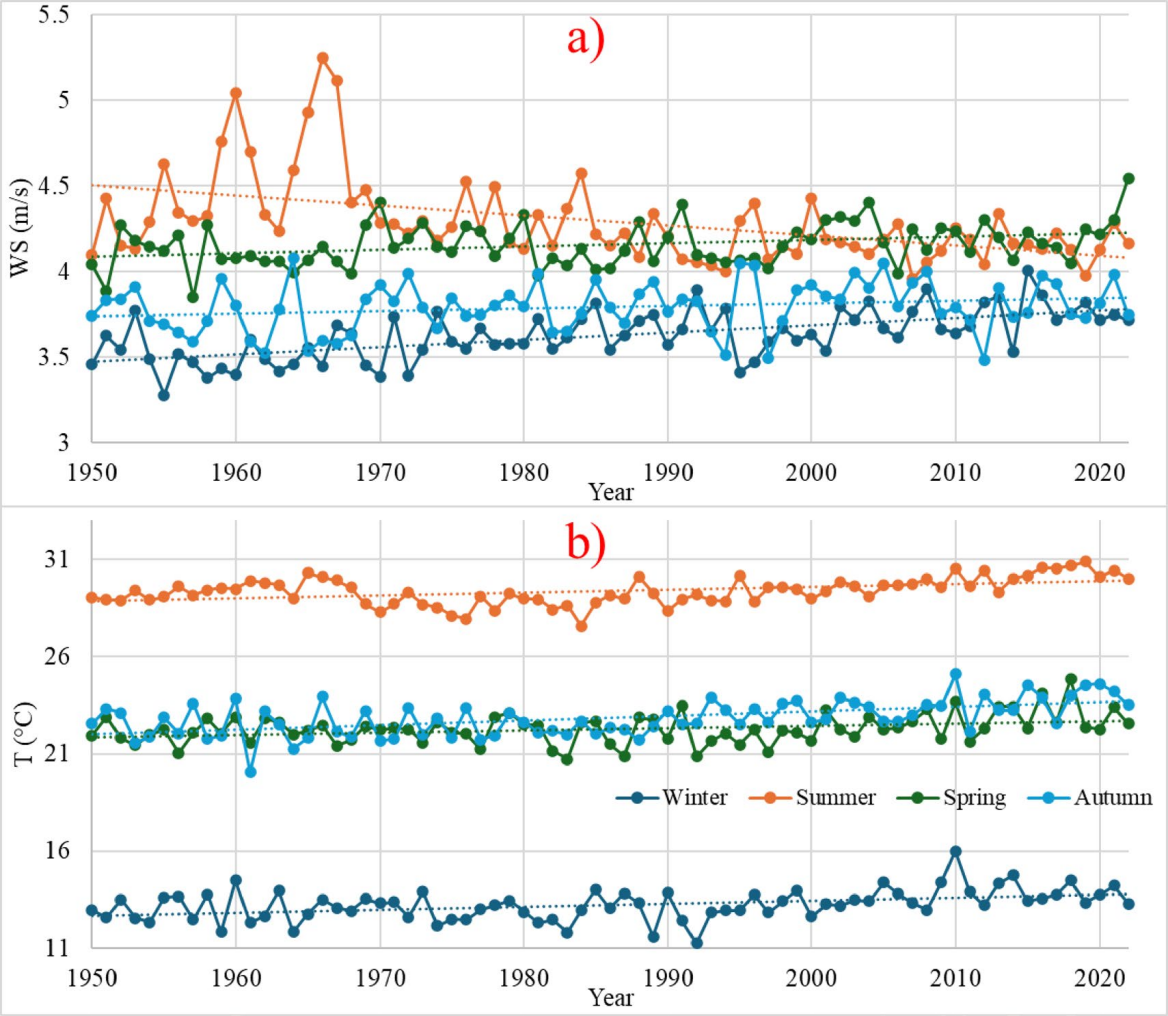
Annual Time series for each season for **(a)** wind speed and **(b)** mean temperature from 1950 to 2022.

Figure 4 presents the seasonal variations and long-term trends of (a) wind speed and (b) temperature from 1950 to 2020, illustrating distinct patterns for winter, summer, spring, and autumn. The seasonal differences in wind patterns during the whole period are shown in Fig. 4. Accordingly, the wind speeds have increased during the winter, spring, and autumn, while slightly decreasing during the summer. Wind speeds are higher in the summer and spring than in the winter and autumn. This demonstrates the seasonal effect for producing wind energy. Furthermore, the relationship between rising temperatures and variations in wind speed provides essential data on the prospective impact of climate change on wind energy supplies, as the same technique is applied to temperature. An increase of one degree is recognized over the last seventy years for each season, as shown in Fig. 4.

**Fig. 5:**
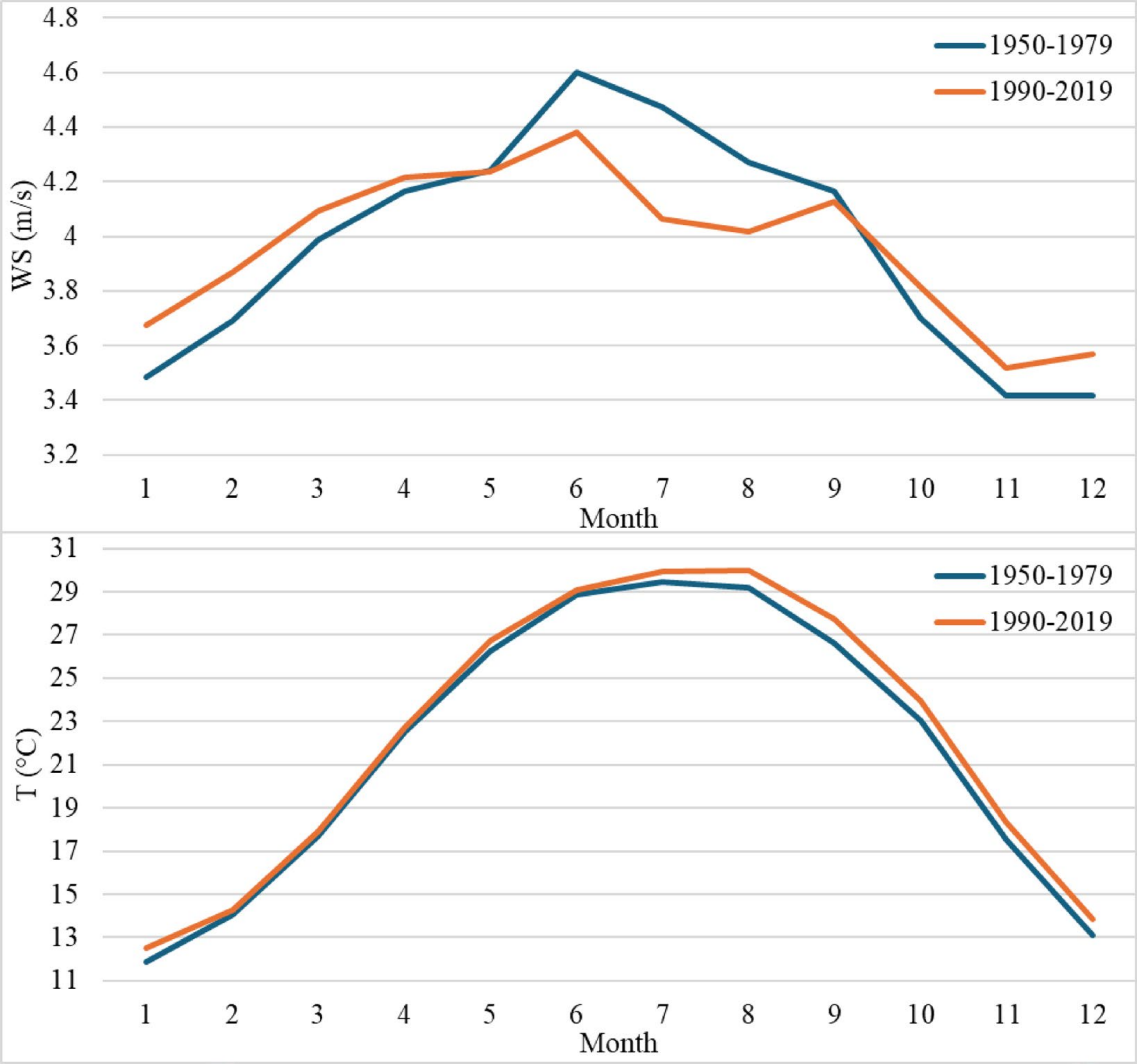
Average monthly time series for **(a)** wind speed and **(b)** mean temperature from 1950 to 1979 (blue line) and from 1990 to 2019 (orange line).

The monthly average time series for period 1 (from 1950 to 1979) and period 2 (from 1990 to 2019) of the wind speed and mean temperature are presented in Fig. 5. During period 1, the maximum wind speed value was 4.6 m/s in June, and the minimum wind speed value was 3.4 m/s, throughout the winter conditions months of November and December. During period 2, the maximum wind speed fell to 4.4 m/s, while the minimum wind speed slightly increased to 3.5 m/s. It characterizes a likely long-term trend toward decreasing extreme winds and points towards an area changing its wind profile. The temperature differential of about 1 °C between the two periods is quite essential since this could impact energy production and wind patterns. The monthly mean temperature of period 2 reached a maximum and minimum value of 30 and 13 degrees Celsius during August and January, respectively. While considering the mean temperature of period 1, the maximum and minimum temperatures were 29 and 12 degrees Celsius, respectively. Increased temperatures can modify the profile of the wind, impacting wind energy systems’ efficiency by altering the stability of the air and, consequently, wind dynamics.

**Fig. 6:**
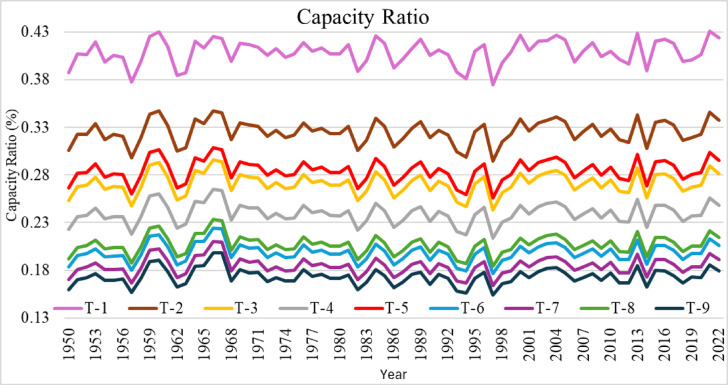
Annual Time series for each wind turbine capacity ratio.

The efficiencies of the nine wind turbines used in this study in a time series manner are presented in Fig. 6. The efficiency of each turbine is detailed over the past 70 years while considering climate change in its performance. As the weather conditions applied to the nine wind turbines were the same, the capacity ratio pattern of all the turbines was similar. On one hand, the study noted that the maximum capacity ratio ratings of turbines T-1 and T-2 range from 38% to 43% and from 30% to 35%, respectively. On the other hand, T-9 and T-7 turbines have very low capacity ratio ratings: 15.3% and 16.2%, respectively. The performance of T-1 and T-2 encourages the view that continuous improvements in turbine design are key to better energy extraction.

**Fig. 7:**
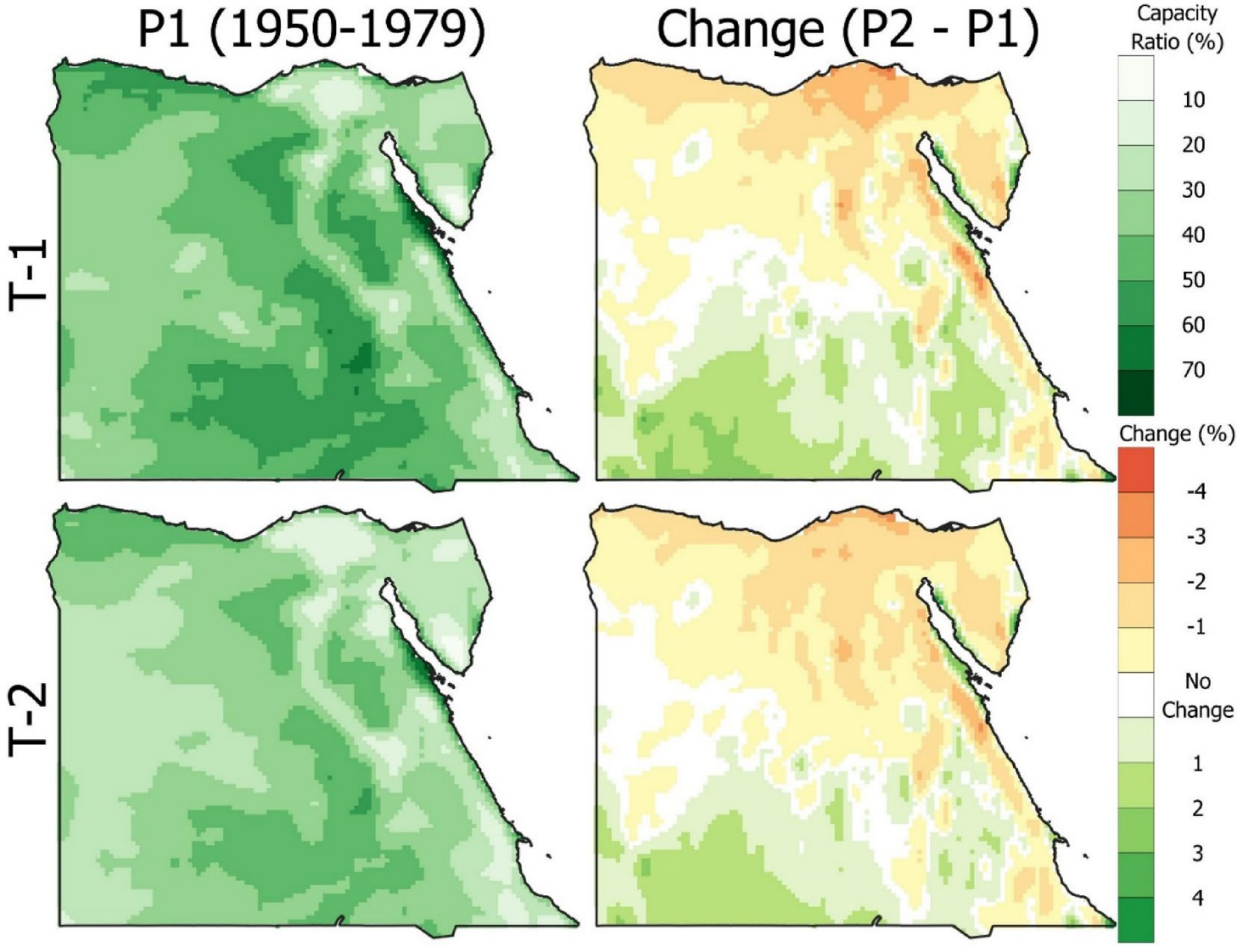
Spatial distribution for wind turbine T-1 and T-2 capacity ratio from 1950 to 1979 (left column) and the change from the reference period (the figure generated using Qgis V3.40 https://qgis.org/download/).

Based on the previous findings, wind turbines 1 and 2 got the highest ratio. The capacity ratio and the change in ratios between periods 2 and 1 for the two turbines are shown in the first and second columns of Fig. 7, respectively. The graphs show differences in ratios, pointing out regions that may cause decreased or increased turbine output due to changes in wind patterns or temperature variation. The contour mapping for the capacity ratio of both turbines, using meteorological data from period 1, reveals that Egypt has high potential for wind turbine installation, particularly in Ras Gharb, due to favorable wind conditions. Also, in the South region, the capacity ratio of both turbine 1 and 2 reached 50% and 45%, respectively. The right column indicates a positive change of 4% and 2% for the capacity ratio of turbines 1 and 2 in the southwest region, respectively. A negative change of 3% is recognized across northern Egypt, with some areas reaching − 4% for both turbines. The current work aims to determine wind turbine operational capacity ratio variations due to climate change. Such a visualization can be made to attract investments in wind energy infrastructure that would be vital in meeting Egypt’s growing demands for energy.

**Fig. 8:**
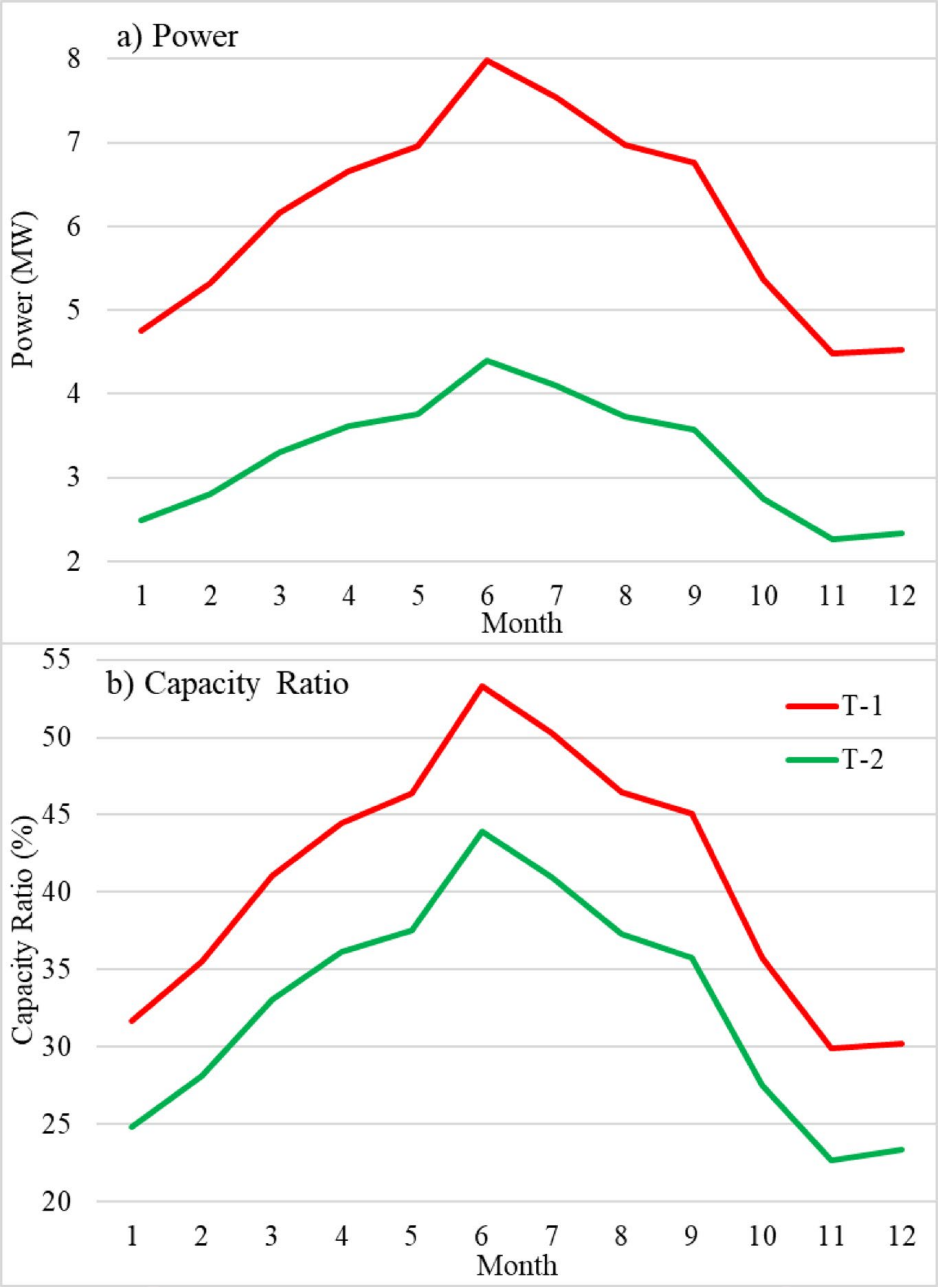
Average monthly **(a)** power and **(b)** capacity ratio time series for wind turbines T-1 (red line) and T-2 (green line) from 1950 to 1979.

A detailed comparison of the power production and capacity ratio of the T-1 and T-2 turbines is shown in Fig. 8, which also highlights the unique operational features of each turbine. Notably, T-1 exhibits an anticipated power output of 8 MW in June and 4.5 MW in November, despite having a rotor radius of 135 m. Meanwhile, T-2 reached maximum and minimum power values of 4.4 MW and 2.3 MW in June and November, respectively. Even with this huge potential for electricity generation, the capacity ratio of T-1 is more than that of T-2. The primary cause of the difference is the direct correlation between power generation is the rotor diameter. Higher power outputs are possible because a larger rotor diameter usually enables greater wind energy capture. However, rotor size may not be the only factor affecting power generation; design considerations, operating conditions, and aerodynamic performance also play a role. Wind turbine 1 achieves a maximum capacity ratio of 53.5% during June, suggesting a more efficient conversion of wind energy into power. Wind turbine 2 reached a maximum capacity ratio of 44% during June.

**Fig. 9:**
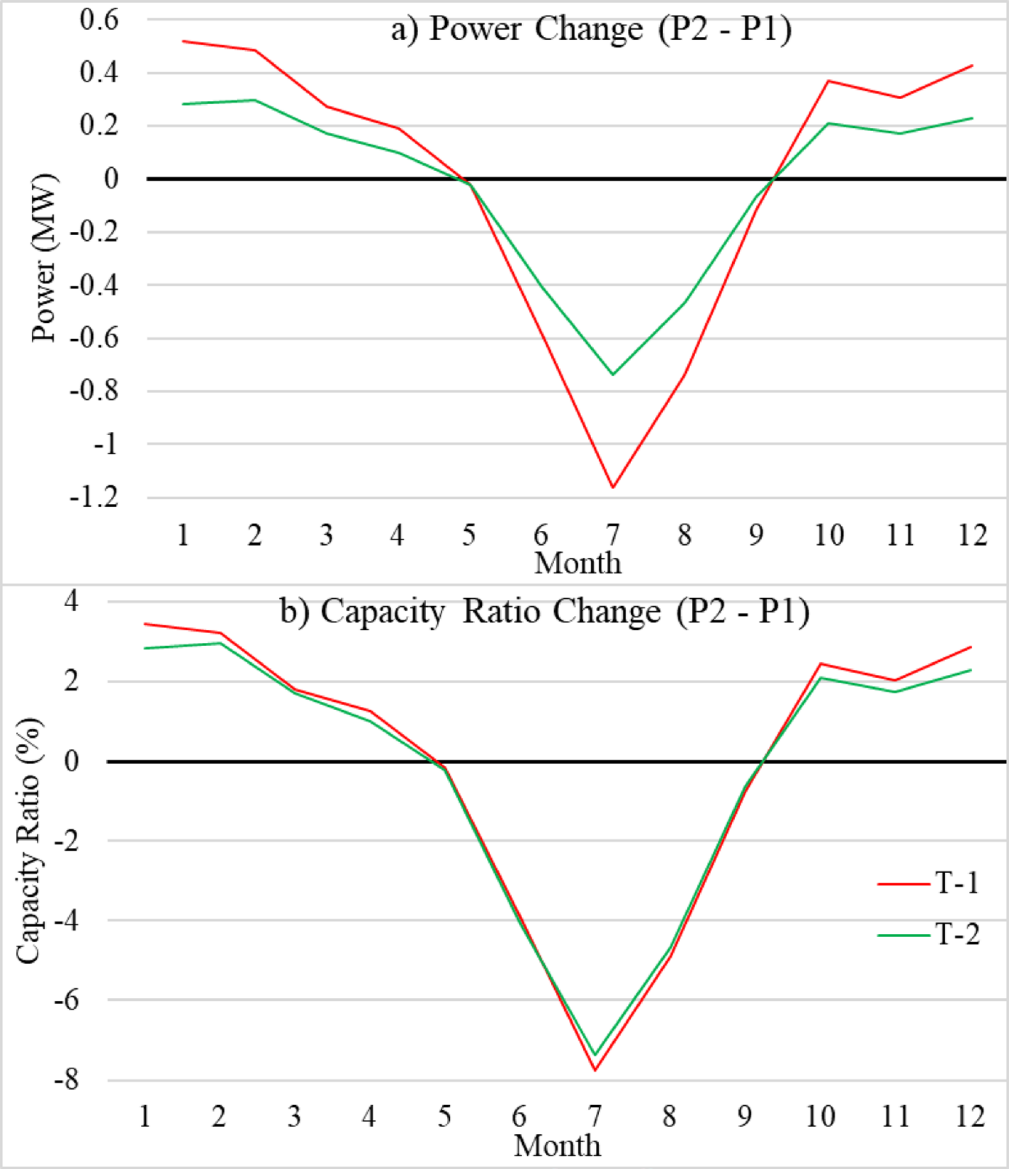
Monthly absolute change (1990–2019) in **(a)** power and **(b)** capacity ratio time series for wind turbines T-1 s(red line) and T-2 (green line) from the reference period.

A monthly time series for wind turbines 1 and 2 shows a change between period 2 and 1 of the output power, and the capacity ratio is presented in Fig. 9. This provides an insightful comparison of the changes in power output and capacity ratio for turbines T-1 and T-2 between period 1 and period 2. A notable decrease in power generation is evident, with T-1 experiencing a reduction of 1.18 MW and T-2 showing a decline of 0.7 MW. This reduction is especially noticeable in July, suggesting a substantial change in operational performance. The data indicate that both turbines’ power production has decreased due to external influences, most likely influenced by the weather. The sensitivity of wind energy systems to environmental changes highlights the necessity for strong planning and management techniques to mitigate the effects of unfavorable weather conditions on energy output. A 7.8% and 7.5% drop in capacity ratio for turbines 1 and 2, respectively. This is closely associated with the drop in power output. The leading cause of this reduction is the effects of climate change, specifically the noticeable drop in wind speed shown in Fig. 5. Turbines’ capacity to produce electricity is always compromised when wind speed decreases, reducing the operational capacity ratio. During February, an increase of 3.2% and 3% in the capacity ratio of both wind turbines 1 and 2, respectively. This led to a rise in the output power by 0.5 and 0.3 MW for wind turbines 1 and 2, respectively. This link highlights the importance of focusing on climate change, as reliable wind patterns are crucial for the feasibility of wind energy generation.

**Fig. 10:**
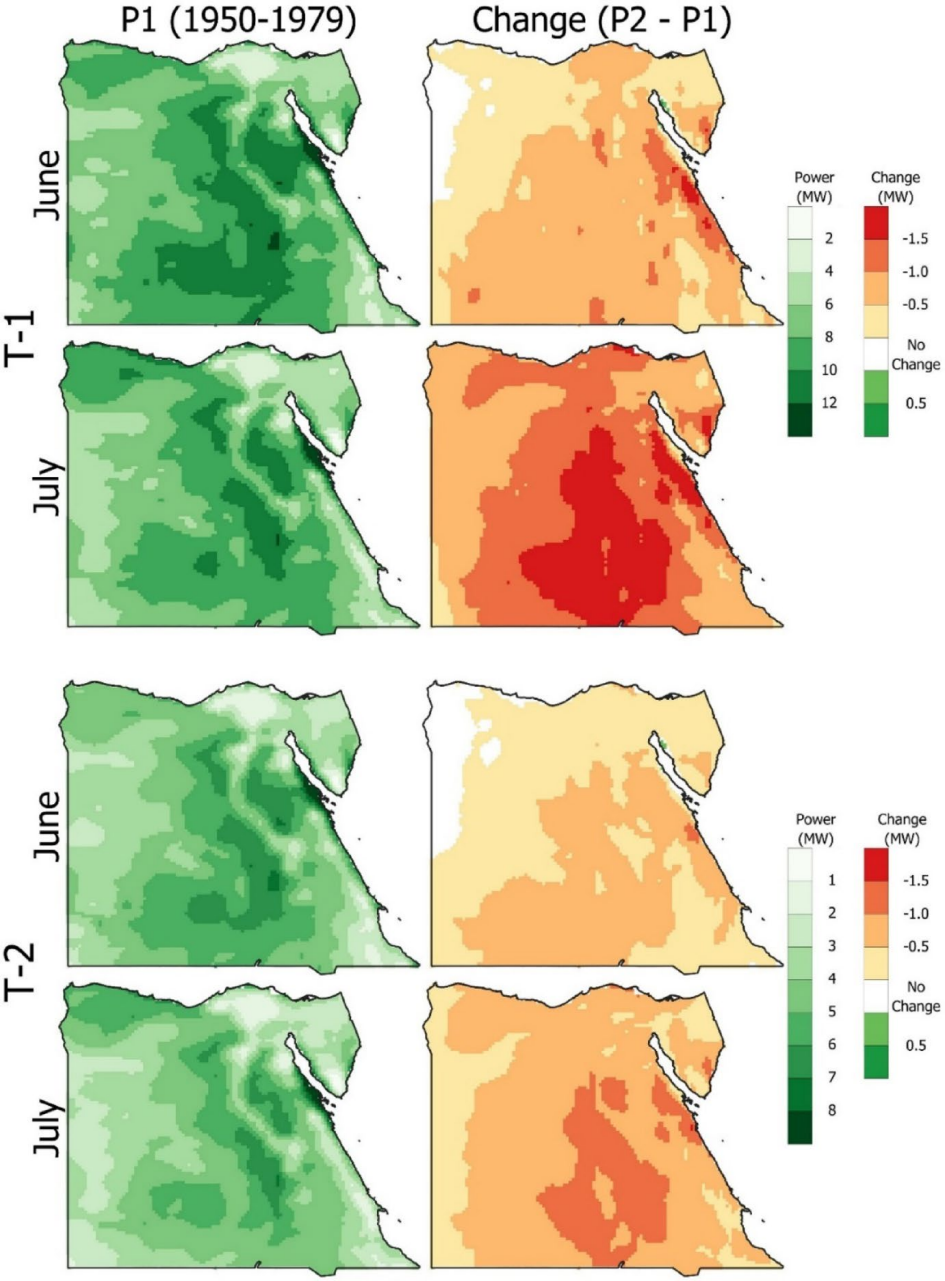
Spatial distribution for output power from 1950 to 1979 (left column) and the absolute change in 1990–2019 compared to the reference period for wind turbines T-1 and T-2 in the highest months (June and July) (the figure generated using Qgis V3.40 https://qgis.org/download/).

The output power during period 1 and its change for both wind turbines 1 and 2 during June and July are presented in Fig. 10. The output power patterns for both wind turbines during June and July, the top output months, are shown in the left column during period 1. Wind turbine (T-1) maximum output power was recognized in Ras Gharb and the middle of Egypt, reaching 12 MW, and high values of 10 MW were recognized in the South. A drop of 1.5 MW was recognized when comparing period 2 to period 1 near the Red Sea during June and across most of Egypt during July. Regarding turbine 2, the maximum output during June and July is 8 MW in Ras Gareb. Across the center of Egypt, the average output power ranged from 4 to 6 MW during period 1. During June, the power change between periods 1 and 2 decreased by 0.5 MW along the Red Sea, while in July, a remarkable drop of 1 MW occurred across most of Egypt.

**Fig. 11:**
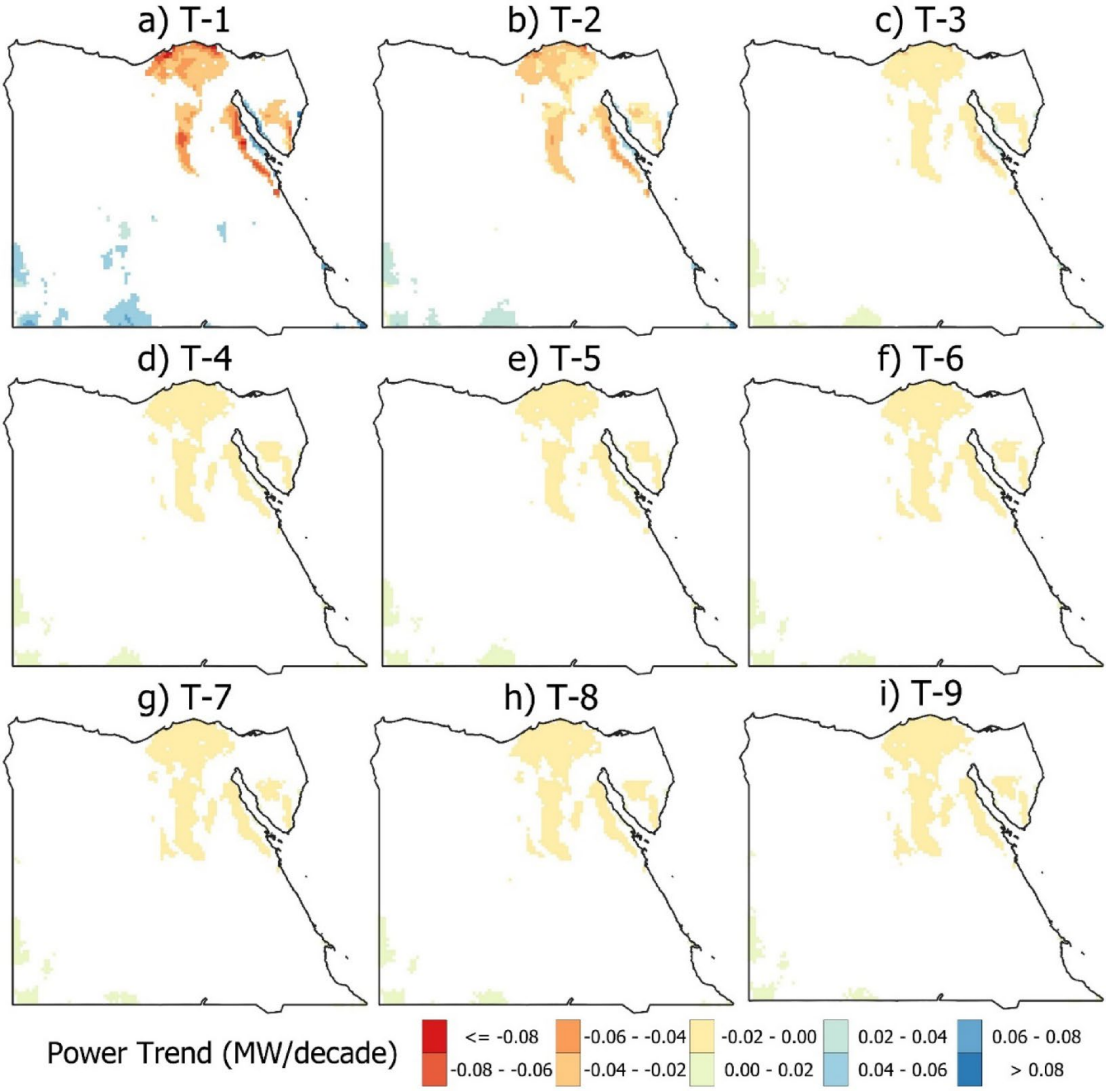
Spatial distribution for significant trend analysis (*P* < 0.05) per decade for power generated from wind turbines T-1 to T-9 (a to i). White areas indicate locations where no significant trend occurred (the figure generated using Qgis V3.40 https://qgis.org/download/).

Figure 11 presents a trend analysis of the output power from nine turbines (T-1 to T-9) in Egypt from 1950 to 2019, using Sen’s slope estimator to quantify the trend magnitude (MW/decade) and the modified Mann–Kendall test to assess statistical significance. The results reveal a clear spatial pattern: all nine turbines exhibited a significant decrease in output power in northern Egypt and along the Red Sea mountains, while an increasing trend was observed in the southwestern region. White areas indicate locations where no significant change occurred. The color ramp is applied only to areas with statistically significant trends. T-1 highlights the contrast between the highest declining ≤ −0.08 MW/decade and the highest increasing > 0.08 MW/decade power trends. The remaining eight turbines (T-2 to T-9) followed a nearly identical trend pattern with very close values, suggesting consistent behavior across these units.

**Fig. 12:**
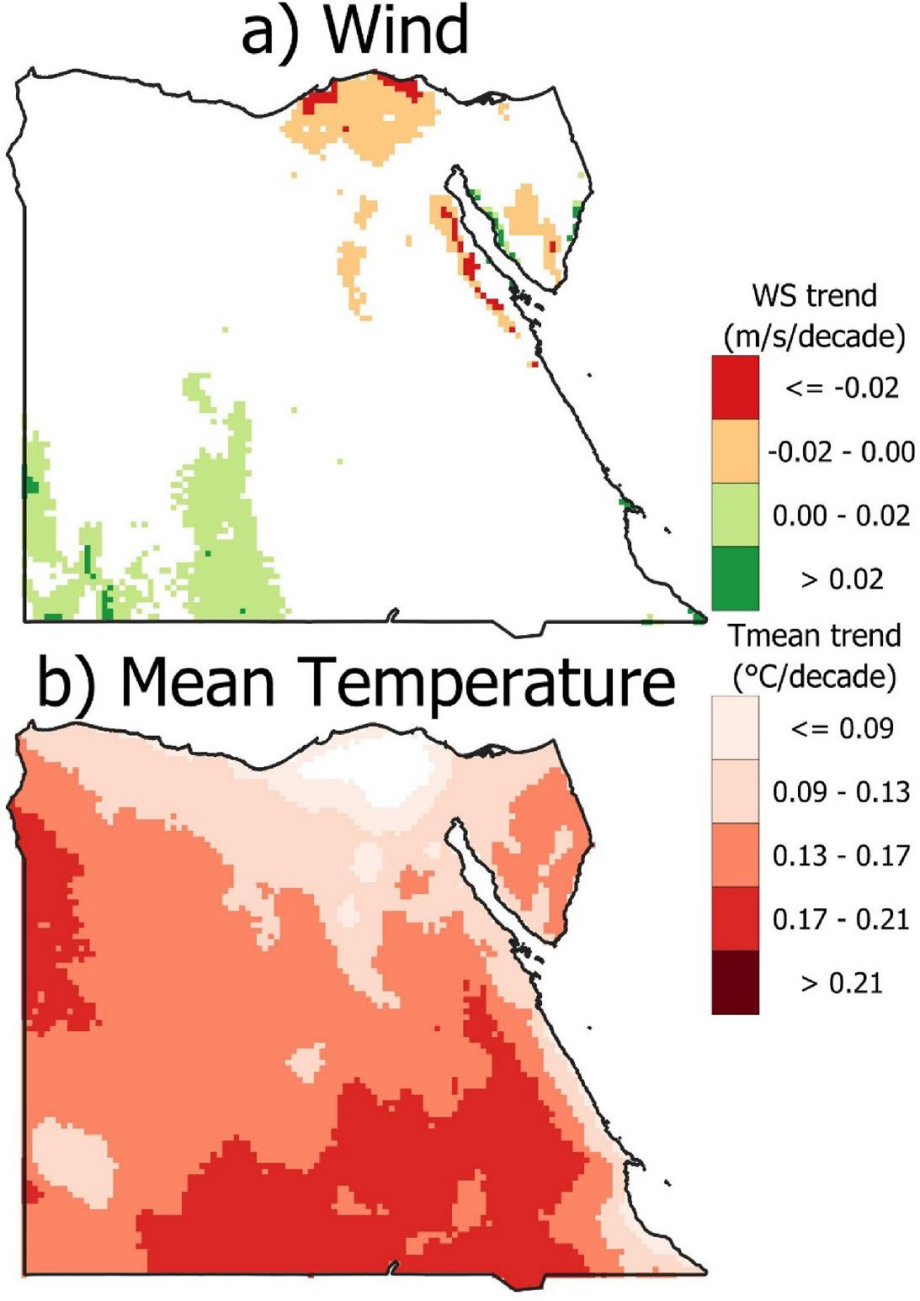
Spatial distribution for significant (*P* < 0.05) trend analysis per decade for **(a)** wind speed and **(b)** mean temperature (the figure generated using Qgis V3.40 https://qgis.org/download/).

The spatial distribution of Egypt for both wind and temperature for significant (*P* < 0.05) trend analysis is presented in Fig. 12. The trend analysis of wind speed across most of Egypt reveals no significant trend. However, there is an increase in the southwest of Egypt with a maximum value of 0.02 m/s/decade and a decrease in the north of Egypt with a value of −0.02 m/s/decade. The same significant trend analysis was applied to the mean temperature. A considerable increase was recognized across all Egypt, except the delta, where the trend was non-significant. An increase in the range of 0.13 to 0.17 and 0.17 to 0.21 °C/decade for the middle and South of Egypt, respectively. The decline in wind speeds (terrestrial stilling) is physically explained by an increase in temperature (0.17 to 0.21 °C/decade) by the following mechanisms: Decreased thermal gradients Near-surface wind speeds are fundamentally driven by pressure gradients, which are caused by differences in temperature (for example: land-sea differences, equator - pole differences or day-night differences). Change in atmospheric circulation Increasing temperature will modify circulation patterns including Hadley Cell expansion or some increased variability of the jet stream reducing important scale mechanisms that produce wind (for example, reduced intensity of storm tracks). Thermal coupling and change associated with Land Surface Change. Increasing temperature will amplify local land-atmosphere feedback (i.e., decrease moisture in surface soil and vegetation, increase roughness) that further dampens wind speeds.

## Discussion

Shifting wind patterns are one of the most significant impacts of climate change on Egypt’s wind energy potential. The scientific agreement is that rising global temperatures affect atmospheric circulation patterns, influencing wind intensity, frequency, and direction^46,47^. In places like Egypt, this could lead to fluctuations in wind velocity and consistency, both critical for the wind turbine capacity ratio. This study employs a thorough methodology to map meteorological conditions across Egypt and evaluate the feasibility of HAWT deployments at various sites. The ERA5 dataset, from 1950 to 2019, provides significant insights into variations in wind speed and temperature in Egypt throughout the decades. This technique enhances the understanding of long-term weather patterns and their impact on wind energy potential throughout different regions of the country.

Using wind data covering the whole Egyptian land was one of the main features of this study’s approach. The computation of wind speed magnitudes by integrating the u and v components at 10 m and adjusting to a 2-meter elevation provides a reliable depiction of wind conditions, a standard reference in meteorological research. This is particularly crucial for HAWT performance, where wind speeds at hub height are critical for efficient energy generation. The data is divided into two periods, 1950–1979 and 1990–2019, allowing for a comparative analysis of temporal changes in weather patterns, particularly wind speeds. Understanding long-term patterns and possible changes in wind energy generating capacity depends on a way to investigate the variability in wind conditions over several decades, which the periodizing provides. Moreover, considering ERA5-land as the main data source brings a degree of accuracy and precision given its global scale, and great temporal and spatial resolution. Regarding turbine choice, the study makes a major contribution by suggesting a location and choice of turbine.

The variations in wind speed and mean temperature across Egypt, comparing P1 (1950–1979) to P2 (1990–2019), as shown in Fig. 3, showed positive and negative changes across different regions. The most significant positive change in wind speed is observed in the southwest of Egypt, while most of the country shows minimal or no change. The Toshka Project is a massive agricultural development project in South-west Egypt, aiming to achieve food security, increase agricultural land, provide job opportunities, and encourage reconstruction in the South Valley region. The promising future of wind energy will support progress.

Wind energy has both economic and social impacts on rural areas, including the effects on the return on investment of wind energy projects, the stability of the power grid, and the local communities. In the present study, the 7.5–7.8% drop in capacity ratios noted between periods P1 and P2 suggests that future projects may face longer payback periods, potentially reducing private investment in regions with declining wind resources. Although from an economic perspective, a decline in a wind farm’s capacity factor directly impacts its revenue stream and consequently its return on investment, wind energy projects can be a catalyst for development, providing clean power for initiatives, creating local jobs, and fostering energy independence.

Also, there is a decrease in the northern coastal region. These results align with earlier research^46^, which attempts to evaluate the effects of climate change on wind and solar energy potential in Egypt by 2065 under the RCP 8.5 scenario compared to the base period (1970 to 2005). In contrast, mean temperature exhibits a consistent positive trend across the country, indicating a general warming pattern. The most pronounced temperature increase is seen in the southern regions of Egypt. These findings highlight the spatial heterogeneity in climate variables, with temperature showing a uniform warming trend, whereas wind speed changes appear more localized and variable.

This study thoroughly evaluates the performance and capacity ratio of nine wind turbines. T-1 and T-2 are the most efficient turbines of all the options. Upon comparison of T-1 and T-2, it was observed that T-1 exhibited superior peak power outputs (e.g., 8 MW in June) followed by T-2 (e.g., 4.4 MW in June). The superior of T-1 is based mainly on the rotor dimensions. Another central finding is the influence of climate change on wind energy viability. Figure 9 illustrates that both turbines significantly reduced power output and capacity ratio between the P1 and P2 periods. A 1.18 MW reduction for T-1, a 0.7 MW reduction for T-2 in July. A 7.8% and 7.5% decline in capacity ratios for T-1 and T-2, respectively. This indicates that fluctuations in wind speed are already affecting electricity generation capacity. Similar trends were observed by^48^, who noted regional wind speed reductions across North Africa, raising concerns about the long-term sustainability of wind energy projects in the region.

Furthermore, this research incorporates multi-period comparisons and turbine-specific performance metrics. This study provides stakeholders with practical insights for strategic turbine placement, specifically following Egypt’s national energy objectives—20% renewable energy by 2030. The comparative analysis of T-1 and T-2 facilitates informed decision-making for turbine selection, ensuring that investments in wind infrastructure emphasize raw production and long-term operating conditions under fluctuating climatic changes. This study underscores the need to incorporate environmental data into turbine planning and implementation. It emphasizes that simply augmenting the turbines’ quantity or dimensions is inadequate. A transition to more intelligent and adaptive technologies is crucial for maintaining the resilience and sustainability of Egypt’s wind energy sector in the context of climate change. Moreover, the impact of temperature on the capacity ratio of wind turbines, while not the central subject of this work, warrants consideration in subsequent research. Elevated temperatures can impact on the mechanical characteristics of materials utilized in turbine fabrication, affecting their long-term performance and maintenance requirements.

The output power patterns for both wind turbines, T-1 and T-2, are shown in Fig. 10, with June and July being the top output months. Both turbines produced their greatest power during the first study period (1950–1979), proving that wind energy generation was effective during these months. It can create a baseline against which performance variations can be evaluated by charting the output power for this historical era. This comparison requires understanding how outside influences, especially those connected to climate change, have affected turbine capacity ratio and power generation over time.

As the investigation moves into period 2, both turbines’ output power has suffered due to a notable decrease in wind speed. This shift is reflected in the statistics, which show that during the peak month of July, T-1 and T-2 significantly decreased by 1.18 MW and 0.7 MW, respectively. This decrease emphasizes how wind energy systems are affected by environmental alterations, especially in wind patterns brought on by climate change^49,50^. This decrease demonstrates how sensitive wind energy systems are to changes in wind speed; even small changes can result in significant drops in power output. Since constant wind patterns are necessary to sustain optimal turbine performance, the consequences of these findings highlight the need for wind energy stakeholders to consider climate change when planning and managing wind energy resources. Furthermore, significant concerns regarding the long-term viability of wind energy in the face of climate change are raised by the noted drops in output power between the two periods. According to the findings, continuous changes in wind patterns may impact wind turbine capacity ratio, requiring adaptive measures to reduce these effects. This requires investment in more sophisticated turbine technology to function well in various climates and installing monitoring systems to monitor wind speed and turbine performance in real time.

## Conclusion and future work

This study advances the understanding of Egypt’s wind energy potential by combining historical climate data with detailed modelling to guide the strategic placement of HAWT. The findings underscore the importance of localized analysis, particularly highlighting the southwest region as a high-potential area for future wind energy projects due to observed increases in wind speed (4.5 m/s). Recently, wind speeds in the South have increased by 0.2 m/s over the past few decades. While the current methodology offers a strong foundation for turbine selection and siting, further research is needed to incorporate additional variables such as grid integration, turbine efficiency, and economic considerations. The results show that the maximum capacity ratio rating of turbines T-1 and T-2 ranges from 38% to 43% and 30% to 35%, respectively. T-1 highlights the contrast between the highest declining ≤ −0.08 MW/decade and the highest increasing > 0.08 MW/decade power trends. The highest wind speed was observed at Ras Gareb (> 5.5 m/s), and an increase of 0.25 m/s compared to the reference period.

Future work should incorporate advanced climate projection models, such as those from the Coupled Model Intercomparison Project Phase 6, to simulate and estimate future wind speed changes under different climate scenarios. This would provide valuable insights into the long-term viability and optimization of wind energy projects in Egypt, enabling better-informed policy and infrastructure planning to the end of the 21 st century.

## Data Availability

Data and code will be available upon request from the corresponding author.
